# Thermal and seismic hints for chimney type cross-stratal fluid flow in onshore basins

**DOI:** 10.1038/s41598-018-33581-x

**Published:** 2018-10-17

**Authors:** Jacques Dentzer, Dominique Bruel, Matthias Delescluse, Nicolas Chamot-Rooke, Laurent Beccaletto, Simon Lopez, Gabriel Courrioux, Sophie Violette

**Affiliations:** 1 UMR 8538, Laboratoire de Géologie, Département de Géosciences, Ecole normale supérieure, PSL Research University/CNRS, 24 rue Lhomond, 75231, Paris, Cedex 05 France; 2 UFR 918, UPMC-Sorbonne Universités, 4 place Jussieu, 75252, Paris, Cedex 05 France; 3 Centre de Géosciences, Mines ParisTech, PSL Research University, 35 rue Saint Honoré, 77305 Fontainebleau, France; 4 Direction des Géoressources, BRGM, 3 avenue Claude Guillemin, BP 36009, 45060, Orléans, Cedex 2 France

## Abstract

When modelling onshore sedimentary basins, modellers generally assume that semi-permeable layers (aquitards) greatly restrict vertical flow between aquifers. Aquitards are therefore considered as confining media and vertical flow is assumed to take place mainly within localised permeable faults, if any. In the offshore context, however, interpretation of seismic data frequently provides evidence of fluid flow between sedimentary layers via structurally disrupted formations (pervasive fractures) recognised as zones of reduced seismic amplitude and generically called “chimneys”. Here we show that chimneys are also present onshore, and that they crosscut confining layers. In the Anglo-Paris Basin, seismic data suggest 1 to 2 km wide zones of disrupted seismic signal spatially correlated to a hitherto unexplained major temperature anomaly of 20 °C. When included in geothermal models using a five-order increase in permeabilities with respect to confining layers, we find that fluid flows vertically through aquifers and confining layers, thereby explaining this major temperature anomaly. Despite the importance of their hydrodynamic and thermal impacts, chimneys – less obvious than faults – have been overlooked as fluid flow paths in many onshore sedimentary basins exploited for their resources. This indicates a clear need for better understanding of pervasive flow paths, especially as the resources and properties of basins (i.e. conventional and unconventional hydrocarbons, geothermal potential, CO_2_ storage, nuclear waste repository, drinking water, etc.) are increasingly being harnessed.

## Introduction

Hydrological regime is one of the main factors controlling temperature in the Earth’s continental crust^1,2^. For example, vertical flows through permeable structures can cause large thermal anomalies in comparison with regimes without fluid flow (i.e. purely conductive thermal regimes). Such anomalies can reach several tens of degrees Celsius, especially within permeable faulted regions^3–6^ in sedimentary basins. Faulted regions cut through aquifers and aquitards, i.e. across reservoirs and confining layers (the latter being also known as semi-permeables).

Such faulted regions and related fractured areas are widely documented in reservoirs thanks to 3D seismic data acquisition for oil^7^ onshore and offshore, and for geothermal^8^ fields in the onshore context.

2D and 3D marine seismic surveys also frequently provide direct evidence for fluid flow paths across reservoirs and confining layers^9–11^ and these paths have been tentatively classified on the basis of their characteristics^10,11^. Some, such as pipes, are strictly columnar. Others, known as chimneys, are large, somewhat irregular zones that extend vertically (^12^ and references therein). Pipes and chimneys – characterised by acoustic blanking (i.e. areas where the amplitude of seismic reflections is low) – are often interpreted as evidence for fluid flow between sedimentary formations via structurally disrupted areas, i.e. via pervasive fractures. Such evidence is, however, rarely reported for onshore basins.

Highlighting fluid flow paths onshore requires a field area where seismic, hydrodynamic and thermal data coverage is dense, as can be the case for fields exploited for hydrocarbons, water or geothermal resources. In this article we focus on the exploited Anglo-Paris Basin, a slowly subsiding intraplate Meso-Cenozoic sedimentary basin overlying a Variscan substratum located in northern France. The concentration of geothermal plants around the city of Paris is one of the highest in the world, with most of the plants exploiting the same formation: the Bathonian (Dogger) aquifer.

**Figure 1:**
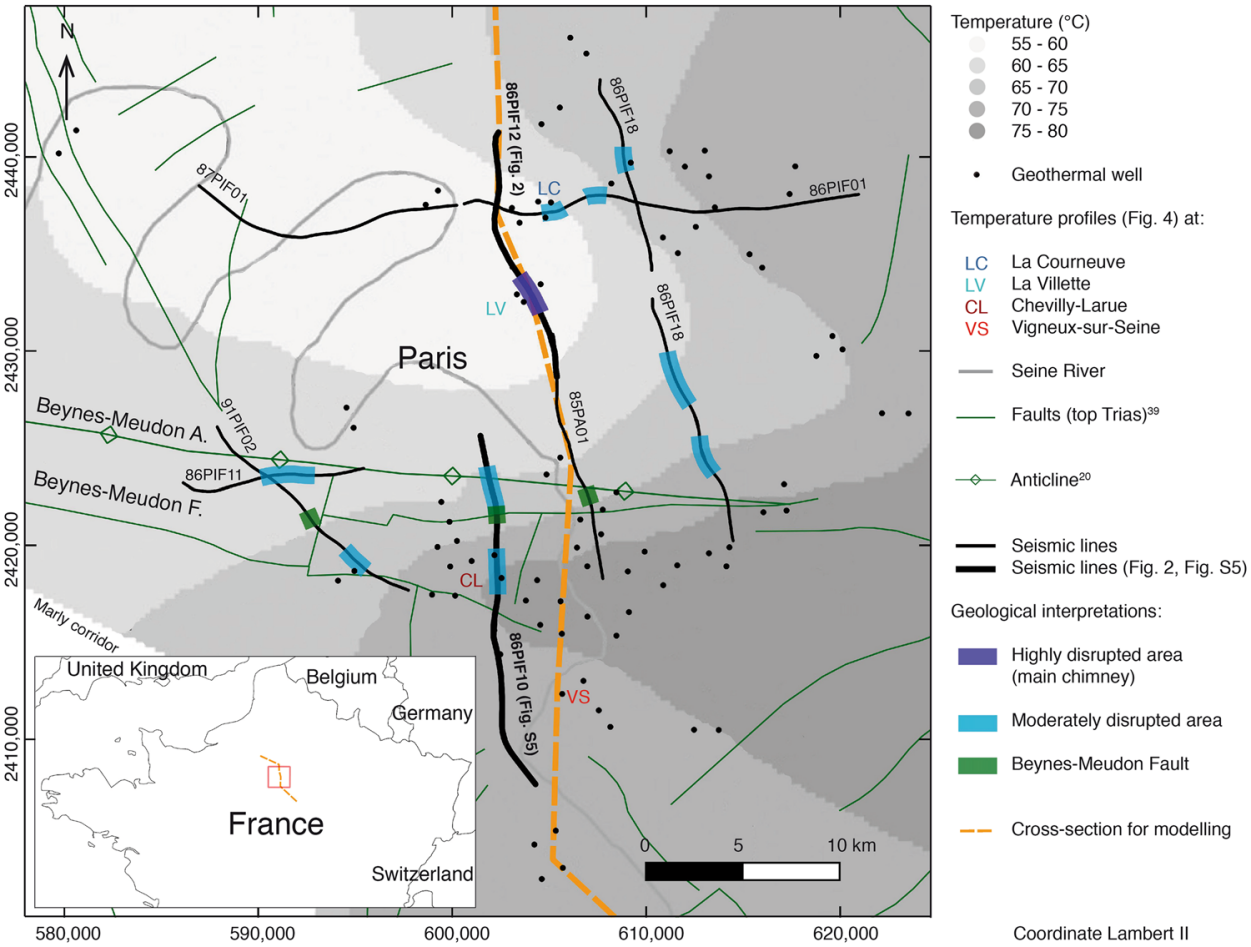
Temperature in the Bathonian and geological structures around Paris in the Anglo-Paris Basin. Overlaying of (i) isotherms (modified from Lopez *et al*.^13^); (ii) locations of temperature profiles illustrated in Fig. 4; (iii) tectonic structures^39^; (iv) proposed geological interpretations (in green and blue) from seismic lines (in black and thicker where illustrated); (v) 2D cross-section (in orange) whose visible section on the main map is the size of Fig. 3.

A 20 °C temperature anomaly is observed within this formation at a depth of around 1,700 m NGF, extending over a few kilometres between the north and the south of Paris^13^ (Fig. 1 and Supplementary Figs S1 and S2). It is colder and less saline in the north and warmer and more saline to the south (Supplementary Fig. S1). No model has so far been able to explain this anomaly, whether it be a conductive model with heterogeneous geothermal flux at the bottom and radiogenic production^14,15^ or an advective one with flow confined to the exploited aquifer^16^.

However, we think that, in spite of its potential significance in terms of thermal and mass transfers, acoustic blanking has been overlooked both in the Anglo-Paris Basin and in many other onshore basins around the world^17^. To date, there has been no systematic search for acoustic blanking in the seismic lines of the Anglo-Paris Basin. We therefore conducted such a search for fluid conduits by carefully screening newly re-processed and re-interpreted seismic profiles. Chimneys were found at several places, a discovery which motivated us to design new models to infer the thermal and hydrodynamic impacts of these features. Building of the thermo-hydrogeological model was also constrained by hydrogeological, geochemical and thermal data.

## Distribution of the Chimneys

We carried out a systematic examination of seismic lines for acoustic blanking (see Supplementary Information and Supplementary Figs S3 and S5) and found areas of reduced seismic amplitude ranging from several hundreds of metres to several kilometres (Fig. 1). We classified these into two categories (“highly” and “moderately” disrupted areas) according to the degree of acoustic signal loss (Fig. 1).

**Figure 2:**
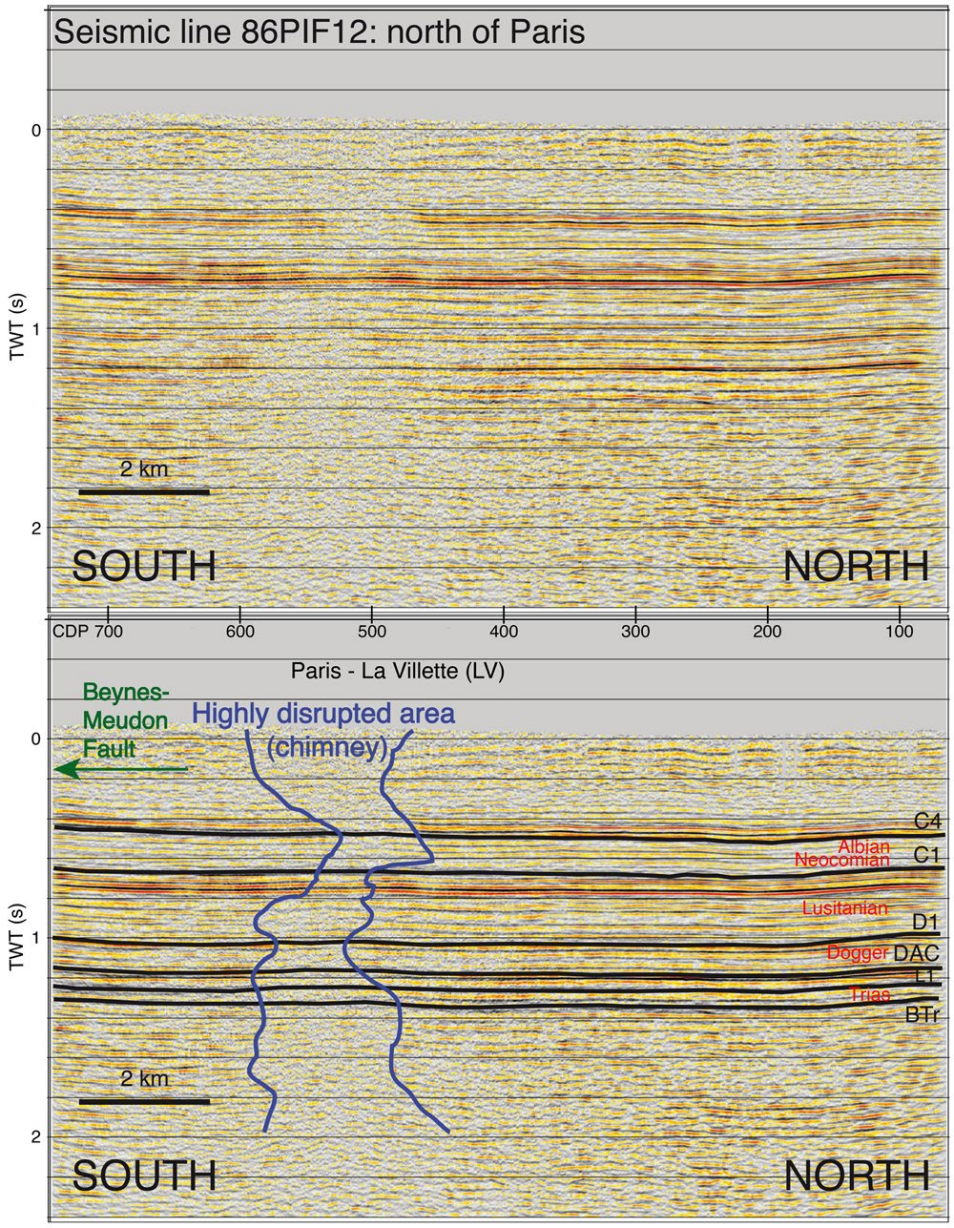
Interpreted north-south seismic line (86PIF12) to the north of Paris (Fig. 1 and Supplementary Fig. S3). Locations of cities in black (Figs 1 and 4). Observation of a chimney: an area with highly disrupted seismic facies bounded by dark blue lines. Main deep aquifers (in red). Levels interpreted (in black): base of the Triassic (BTr); top of Triassic (L1); top of marls at Ostrea Acuminata (Dac); top of limestone Dogger which is top of Dalle Nacrée (D1); lower Berriasian/upper Berriasian limit (C1); and top of Albian (C4).

The highly disrupted area (in dark blue) is shown on a north-south seismic line in the north of Paris (Fig. 2, track line located in Fig. 1 and Supplementary Figs S3 and S4). This line shows a more or less tabular sequence of highly reflective horizons interrupted by an area with a low-amplitude, semi-transparent signal. The shape of this blanking area on the north-south seismic line in the north of Paris (Fig. 2) is irregular at depth. Its width fluctuates between 800 and 2,800 m and its height is over 2 km for the inner, most disrupted area (in dark blue). Reflections within this disrupted area seem to be slightly upwarped.

Several other moderately disrupted areas were found, particularly in the south, albeit with signal attenuation less severe than in Fig. 2 in the north of Paris. These are mainly around the Beynes-Meudon fault-anticline system (Fig. 1, Supplementary Information and Supplementary Figs S3 and S5).

In the Supplementary Information we provide further information about acquisition and processing of the dataset to conclude that – although it cannot be ruled out entirely – it is unlikely that these zones are artefacts. We interpret these areas rather as chimneys.

This distribution of chimneys led us, initially, to integrate the documented thermal and hydrodynamic context. The potential thermal and hydrodynamic impacts of the chimneys were tested by building a chimney scenario, described below. The distribution and possible origin of the chimneys, often explained by hydraulic fracturing, are discussed in a subsequent section.

## Building a Chimney Scenario

Fluid flow within vertically extending permeable fractured zones in the north of Paris was previously unknown, but the possibility was considered to explain temperature variations^15^. The regional temperature trend (Supplementary Fig. S2) suggests that the north of Paris, where the main chimney is located (Figs 1 and 2 and Supplementary Fig. S3), is abnormally cold. We therefore assumed as a first approach, a simplified scenario where this thermal anomaly relates to vertical flow into this main chimney (Fig. 2). This will be discussed later. Flow across the confining layers via the chimney is allowed, between the Triassic, Bathonian, Lusitanian, Neocomian and Albian aquifers. This chimney corresponds to a vertical permeable zone in the 2D model (see Methods and Supplementary Information for more details).

The 2D thermo-hydrogeological numerical modelling included conduction, advection and convection. Advection is due to topography-driven flow, as variations in topography create hydraulic head gradients. Hydraulic head is a combined measure of elevation and water pressure at a given point in an aquifer (see Methods). Convection is due to density-driven flow which, in this model, is caused by thermal variations (see Methods and Supplementary Information for more details). The coexistence of advection and convection corresponds to mixed convection, which was integrated into the modelling process.

Models with (Fig. 2) and without a chimney were explored. All simulations comprised two phases of calculation (1 and 2). A steady-state conductive regime (phase 1) was used to initialise a 5 Ma transient mixed-convective regime (phase 2) that attains a pseudo-equilibrium (for further details, see Methods).

## Results

### A large thermal anomaly reproduced by chimney fluid flow

**Figure 3:**
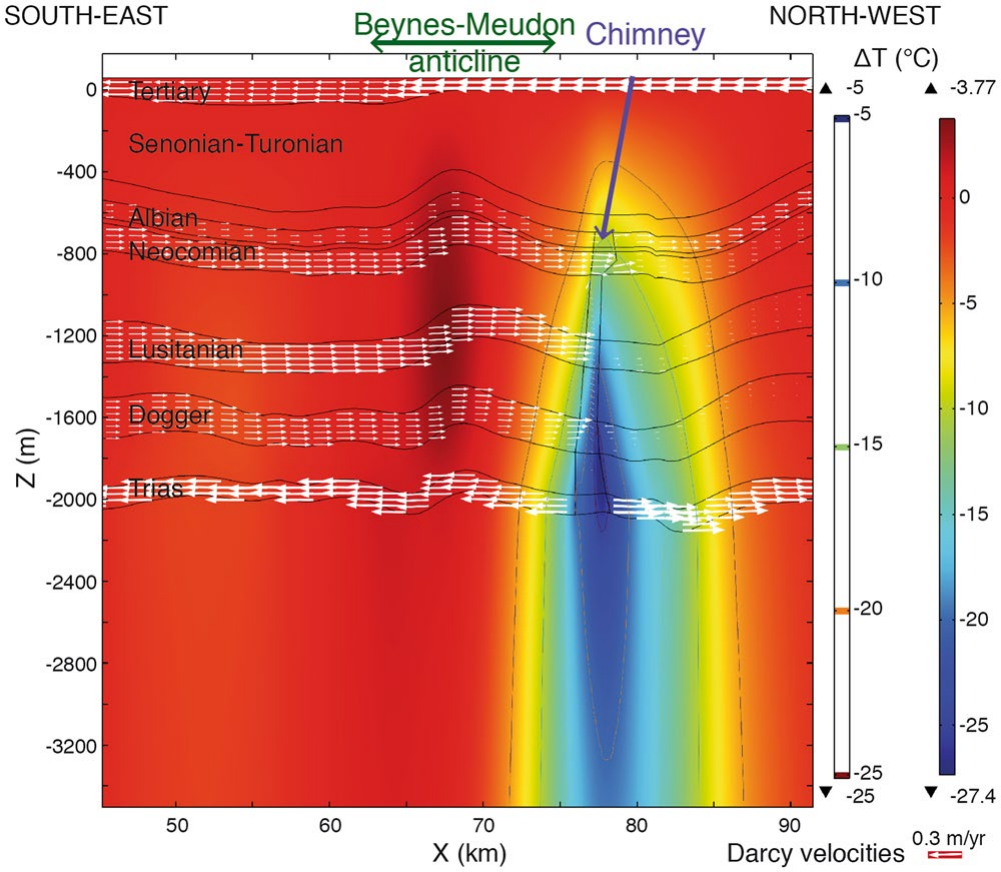
Thermal contribution in °C from mixed convection within the chimney (Fig. 2) and geological formations (for localisation, see Fig. 1 whose visible cross-section on the main map is the same size as Figs 3 and 5). Difference between temperatures at the end of phase (2) with mixed convection (5 Ma) and of phase (1), conductive (steady-state). The Darcy velocities are represented in terms of their directions and amplitudes (white arrows). After sampling at kilometres 50, 70 and 90, Darcy velocities in the aquifers are around 0.03 m/yr to 0.45 m/yr.

**Figure 4:**
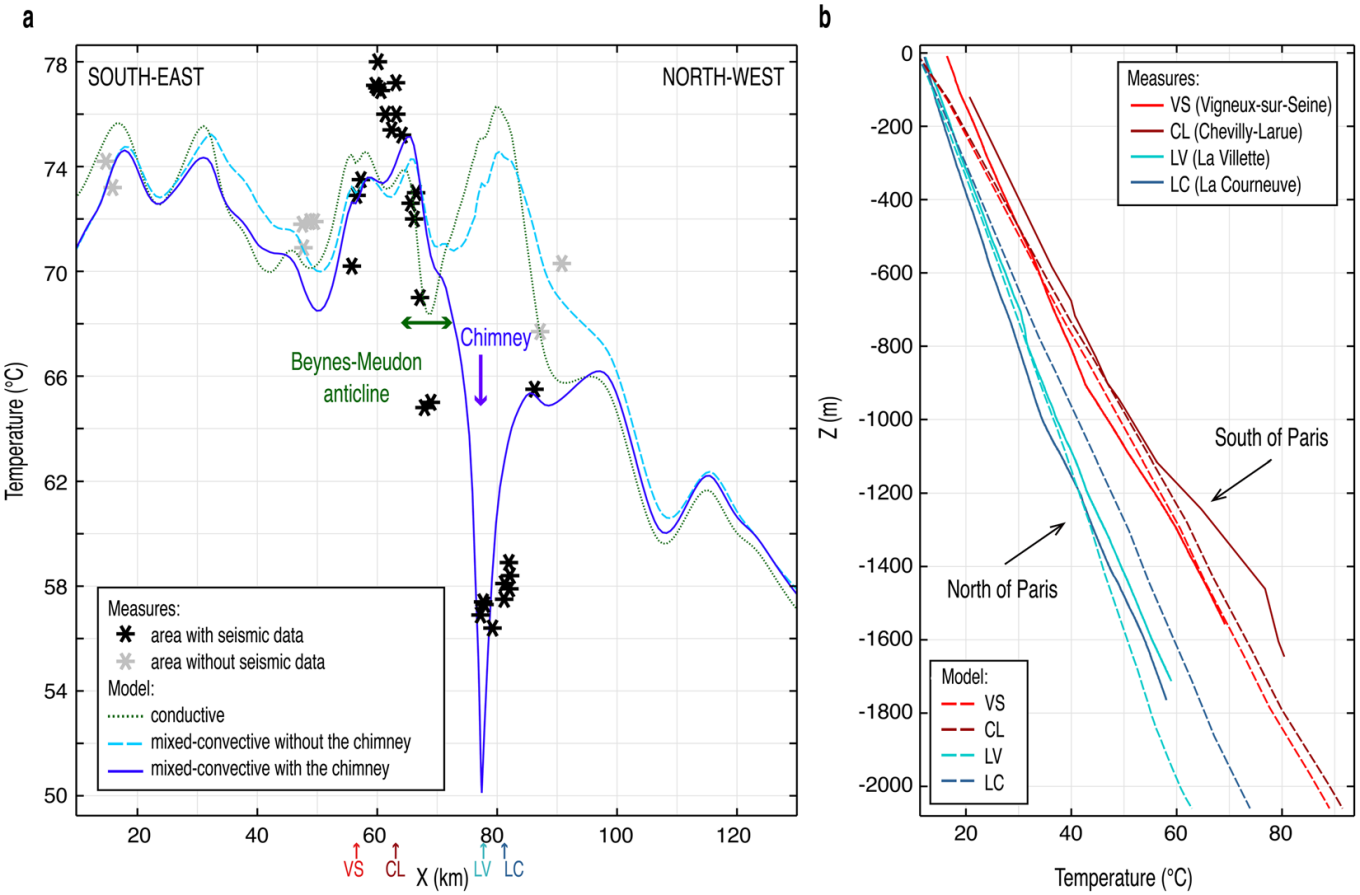
Comparison of simulated and measured temperature in the Bathonian (Dogger) aquifer and at temperature profiles. (**a**) Temperature (top of the Dogger aquifer) of phase (1), conductive and at the end of phase (2) with mixed convection (5 Ma) without and with a chimney (Fig. 2) and temperature measurements in geothermal wells. (**b**) Temperature at end of phase (2) with mixed convection (5 Ma, dashed line) and measured temperature profiles at Vigneux-sur-Seine^15^ (VS) (Fig. 1), at Chevilly-Larue^35^ (CL) (derived from mean gradients per formation), at La Villette^15^ (LV) and La Courneuve^15^ (LC) (unbroken line).

Our findings show that a large thermal anomaly in a basin is reproduced if vertical fluid flow through a chimney, and thus through the confining layers, is considered (Fig. 3). This anomaly is not explained by a conductive approach (green dotted line in Fig. 4) nor by a classic mixed-convective approach confined mainly to aquifers by confining layers: i.e. without a chimney (light blue, dashed line in Fig. 4). However, with a chimney, the simulated convective thermal anomaly reaches −27.4 °C in comparison to a conductive regime (Fig. 3 and dark blue solid line in Fig. 4).

A permeable chimney is therefore seen to have strong hydrodynamic and thermal impacts in a sedimentary basin. A part of the flow moves downward within the chimney, by advection in the lower aquifers. The hydraulic head in the upper aquifers is higher than that in the lowest aquifer, which is the Triassic aquifer (Table 1). Consequently, this downward flow cools the lower layers progressively in comparison to a conductive regime (Fig. 3).

**Table 1:** Properties and boundary conditions of geological formations/units and structures.

Hydrogeological units/formations	Porosity (%)	Intrinsic Permeability (m^2^)	Specific storage (1/m)	Thermal conductivity (W/m/K)	Hydraulic heads (m) (SE - NW)
Tertiary	25	4.5 × 10^−12^	3.2 × 10^−6^	1.6	50–100
Senonian-Turonian	28	5.0 × 10^−15^	2.0 × 10^−6^	1.9	50–100
Albian shale, Cenomanian	20	3.2 × 10^−20^	3.8 × 10^−6^	1.9	
Albian	30	8.0 × 10^−13^	3.8 × 10^−6^	1.7	142–92
Aptian	25	4.0 × 10^−20^	2.3 × 10^−6^	1.7	
Neocomian	28	8.0 × 10^−13^	4.5 × 10^−6^	1.8	162–112
Kimmeridgian, Portlandian, Purbeckian	14	2.0 × 10^−18^	2.1 × 10^−6^	2.1	
Lusitanian	15	8.0 × 10^−13^	2.2 × 10^−6^	2.2	167–128
Callovian, Oxfordian	13	2.9 × 10^−19^	1.7 × 10^−6^	2.0	
Bathonian	16	8.0 × 10^−13^	1.5 × 10^−6^	2.1	172–144
Lias, Toarcian, Aalenian, Bajocian	13	4.4 × 10^−19^	4.2 × 10^−7^	1.6	
Undifferentiated triassic formations	14	8.0 × 10^−13^	3.8 × 10^−7^	1.8	160–105
Bedrock	10	4.0 × 10^−20^	1.0 × 10^−7^	1.9	
Chimney (fractured zone)	20	5.0 × 10^−14^	1.0 × 10^−6^	1.9	

The mixed-convective cold thermal anomaly is established in terms of amplitude and extent from 1.8 Ma of simulation and is stable thereafter. In fact, the anomaly evolves during the 5 Ma transient mixed-convective regime (phase 2) from −8.2 °C at 10 ka to −20.7 °C at 95 ka and −26 °C at 750 ka. It propagates conductively, especially to the deeper horizons, and laterally. The lateral extent of the less than −5 °C anomaly is 16 km in the north of Paris (Fig. 3).

With a chimney, the simulated lateral temperature difference between the south and the north of Paris increases over depth to more than 20 °C, which better explains the thermal regime (Fig. 4a,b). Although 2 to 4 °C are missing in the south of Paris (dark blue solid line in Fig. 4a), the simulation reproduces the temperatures to the south fairly faithfully (Fig. 4a,b), especially at Vigneux-sur-Seine (VS), 20 km from the chimney (Figs 1 and 4b and Supplementary Fig. S1). The abnormally cold temperatures to the north of Paris are then approximated with a minimum extreme temperature of 50 °C (Fig. 4a). Even if the temperatures are not reproduced exactly, they are approximated for La Villette (LV), 1 km from the chimney (Figs 1 and 4b and Supplementary Fig. S1), and La Courneuve (LC), 4 km from the chimney (Figs 1 and 4b and Supplementary Fig. S1) in the scenario presented.

In numerous alternative scenarios studied but not presented, permeability is one of the critical parameters. The cold anomaly appears from a permeability value of 2 * 10^−16^ m^2^ in the chimney. It is about −6.5 °C for 5 * 10^−16^ m^2^, colder than −10 °C for 10^−15^ m^2^ and finally about −23.8 °C for 10^−14^ m^2^, which is of the same order of magnitude as the scenario presented. We also tested higher permeabilities in the chimney to the north of Paris. These alternative scenarios could cause natural convection with convection cells in the chimney, as convection cells lead to downward and upward fluid flow. The latter results in a warm thermal anomaly to the north of Paris, which contradicts the known thermal data. Upward fluid flow from the Triassic, which is more saline, would also contradict the geochemical data (see Supplementary Information for modelling perspectives).

## Discussion

### Origin of the chimneys

As no or few examples of such fluid flow paths are currently known in an onshore context, we compared our observations to offshore examples, since pipes and chimneys have, to date, been observed mainly in offshore contexts^18^. There is, however, one exception in terms of potential analogues for small outcropping pipes, on a shore of the island of Rhodes (Greece), down-scaled in comparison to other offshore observations^19^ studied. Conversely, our main observation in the north of Paris is of large and somewhat irregular features that are not strictly columnar, as are pipes. They can therefore be classified under the generic term of “chimney”^12^. Fairly similar chimneys have been found, for instance, within the Viking Graben. In particular, there are large and irregular chimneys corresponding to type-B in the nomenclature established by Karsten *et al*.^12^.

The mechanisms of formation of such pipes and chimneys remain open to question. Different mechanisms for pipe formation are considered^18^, including hydraulic fracturing. Although karstification could also be considered, since chimneys do cross carbonated formations, they also cross permeable sandstone and several low permeability marly or argillaceous formations, meaning that karstification cannot be regarded as the sole explanation. Mechanisms may coexist or may serve as initiation or growth mechanisms^18^. For instance, according to Løseth *et al*.^19^, repeated episodes of hydraulic fracturing by an incompressible fluid may cause irregular shapes. The irregular and large type-B-chimneys in the Viking Graben have, for example, been associated with a less rapid formation than other chimney types and a gas-dominated flow, according to Karsten *et al*.^12^.

Several types of forcing such as tectonics, sedimentation or paleoclimatic variations may also interact.

First, folding is well documented in the Anglo-Paris basin, as evidenced by the Beynes-Meudon anticline^20,21^. This fold is bordered by the E-W northward dipping Beynes-Meudon fault to the south (see Supplementary Fig. S5). The fault roots into the ante-Triassic substratum and cuts through the Triassic and Jurassic formations. A displacement of these horizons on either side of the fault is, in fact, observed. It is worth noting that some observations are in the south of Paris close to the fault-anticline system. They may therefore relate to fractured areas in the vicinity of faults. The Beynes-Meudon fault probably acquired its present-day reverse-fault geometry during the N-S compressive Pyrenean phase, which started in the Late Cretaceous and peaked in the Late Eocene^20^.

Second, the period from the end of the Cretaceous to the present day is also marked by formational and cross-formational hydrocarbon migration in the basin. For instance, oil was found in the Beynes-Meudon anticline in the Triassic^22^.

Lastly, paleoclimatic forcing is clearly an issue. Transient paleoclimatic forcings are responsible for a transient hydrodynamic regime. The development of permafrost during glacial periods reduced permeability and interrupted recharge^23^. As the basin is drained at the English Channel but not recharged, the hydraulic heads decreased during the glacial periods^23^ and vertical flows were modified accordingly, depending on the rate of draining and recharge of each aquifer. Vertical flows were reversed episodically during the last 100 ka glacial-interglacial cycles and pressure perturbations occurred due to permafrost development^24^. These modifications of hydrodynamic forcings have certainly interacted with the heterogeneities inherited from previous tectonic phases, such as anticlines and synclines.

### Strong heterogeneities of transfers and interrelated exploitations

Subsurface geo-plumbing and seal bypass systems^10^ need to be considered onshore as well as offshore. Onshore basins can no longer be regarded simply as multi-layered systems with reservoirs that are disconnected except at permeable faults. Before considering “confinement layers” as confining media, there is a need to better describe fluid flow conduits by including the chimneys and pipes that may be indicated thanks to investment in 3D seismic surveying. These types of structures are discreet compared to faults with displacements which are easily detectable in seismic profiles. As a result, chimneys and pipes, and their hydrodynamic and thermal impacts, may have been overlooked^17^ so far in many exploited onshore sedimentary basins, despite the capital importance of explaining thermal variations in such basins for their geothermal exploration and exploitation (i.e. for heating and electricity production).

Onshore sedimentary basin formations are increasingly being exploited and use conflicts are already becoming an issue. Basins are harnessed, or are planned to be harnessed, for their natural resources and properties, e.g. conventional and unconventional hydrocarbons; CO_2_ storage; geothermal resources; nuclear waste repository; or, more specific to the onshore context, drinking water. In the case of the Anglo-Paris Basin, one of the aquifers crossed by the chimney is used for drinking water. A large piezometric cone centred on Paris^25^ is observed in the Albian aquifer, due to drawdown. This aquifer is considered to be a strategic reserve in case of a natural disaster or nuclear accident with release of radioactivity.

## Methods

### Numerical modelling

Fluid flow and heat transfers in the model were solved by numerical modelling. Where the geological formations and chimney are concerned, the medium was considered to be porous and saturated. The properties of water *ρ*_*w*_, *μ*, *λ*_*w*_ are those of pure water and are temperature dependent. Dependence on salinity is not included but its hydrodynamic effect is discussed (Supplementary Information).

Fluid flow is assumed to be Darcean. It is described by the diffusivity equation (1), which is derived from the equation for the conservation of mass and Darcy’s law (2) (terms of the equations are given in a list in Supplementary Information and tensor quantities are in bold):

1 $${\rho }_{w}(\,\frac{{S}_{s}}{{\rho }_{w}g}\,)\,\frac{\partial p}{\partial t}+{\nabla }.({\rho }_{w}{\bf{U}})=0$$

where *ρ*_*w*_ is the density of water, *S*_*s*_ the specific storage coefficient, *g* the gravity, *p* the pressure, t the time, and **U** is the Darcy velocity;

2 $${\bf{U}}=-\,\frac{k}{\mu }({\nabla }p+{\rho }_{w}\,g{\nabla }z)$$

where *k* is the intrinsic permeability, *μ* the dynamic viscosity, and *z* the spatial dimension.

The energy conservation equation describes heat transfers (3): convective transfer, Fourier’s law for conductive transfer (4) with inclusion of dispersive phenomena (5, 6) and without taking into account heat production in the domain;

3 $$\frac{\partial (\rho {C}_{p}\theta )}{\partial t}+{\nabla }.({\rho }_{w}\,{C}_{pw}\theta {\bf{U}})+{\nabla }.{\boldsymbol{\varphi }}=0$$

where *ρ* is the saturated matrix density, *C*_*p*_ the saturated matrix heat capacity, θ the temperature, *C*_*pw*_ the heat capacity of water, and **ϕ** is the heat flux density;

4 $${\boldsymbol{\varphi }}=-\,{{\boldsymbol{\lambda }}}_{{\bf{e}}{\bf{q}}}{\nabla }\theta $$

where **λ**_**eq**_ is the equivalent thermal conductivity;

5 $${{\boldsymbol{\lambda }}}_{{\bf{e}}{\bf{q}}}=\lambda {\bf{I}}+{{\boldsymbol{\lambda }}}_{{\bf{d}}{\bf{i}}{\bf{s}}{\bf{p}}}$$

where *λ* is the saturated matrix thermal conductivity, **I** the identity matrix and **λ**_**disp**_ is the macrodispersivity term;

6 $${{\boldsymbol{\lambda }}}_{{\bf{d}}{\bf{i}}{\bf{s}}{\bf{p}}}=\frac{\,{\rho }_{{\rm{w}}}{C}_{{\rm{pw}}}}{|{\bf{U}}|}[\begin{array}{cc}{\alpha }_{{\rm{l}}}{{{\rm{U}}}_{X}}^{2}+{\alpha }_{{\rm{t}}}{{{\rm{U}}}_{Z}}^{2} & ({\alpha }_{{\rm{l}}}-{\alpha }_{{\rm{t}}}){{\rm{U}}}_{X}{{\rm{U}}}_{Z}\\ ({\alpha }_{{\rm{l}}}-{\alpha }_{{\rm{t}}}){{\rm{U}}}_{X}{{\rm{U}}}_{Z} & {\alpha }_{{\rm{t}}}{{{\rm{U}}}_{X}}^{2}+{\alpha }_{{\rm{l}}}{{{\rm{U}}}_{Z}}^{2}\end{array}]$$

where *α*_*l*_ is the longitudinal dispersivity and *α*_*t*_ is the transverse dispersivity.

These flow and heat transport equations were solved with the COMSOL Multiphysics® software in the 2D model, which uses the finite element method. The geometry of this model was discretized by an unstructured triangular mesh of variable size. Distribution was from finest elements within the chimney to coarser elements in the substratum. As a result, the mesh was around 50,000 mesh elements; a finer mesh was tested but did not alter the results.

### Properties of the geological environment

Homogeneous properties are attributed to the chimney and to each geological formation (Table 1).

Permeability is a critical parameter, as advection and convection depend on flow velocity. It is a parameter that becomes even more critical when a new geological object is considered in a sedimentary pile. Intrinsic permeabilities initially issue from, or are calculated on the basis of, data in the literature for aquitards^25^ and the following aquifers: the Tertiary^26^, the Chalk^27^, the Albian^27^, the Neocomian^27^, the Lusitanian^28^, the Bathonian^29^ and the Triassic^30^.

**Figure 5:**
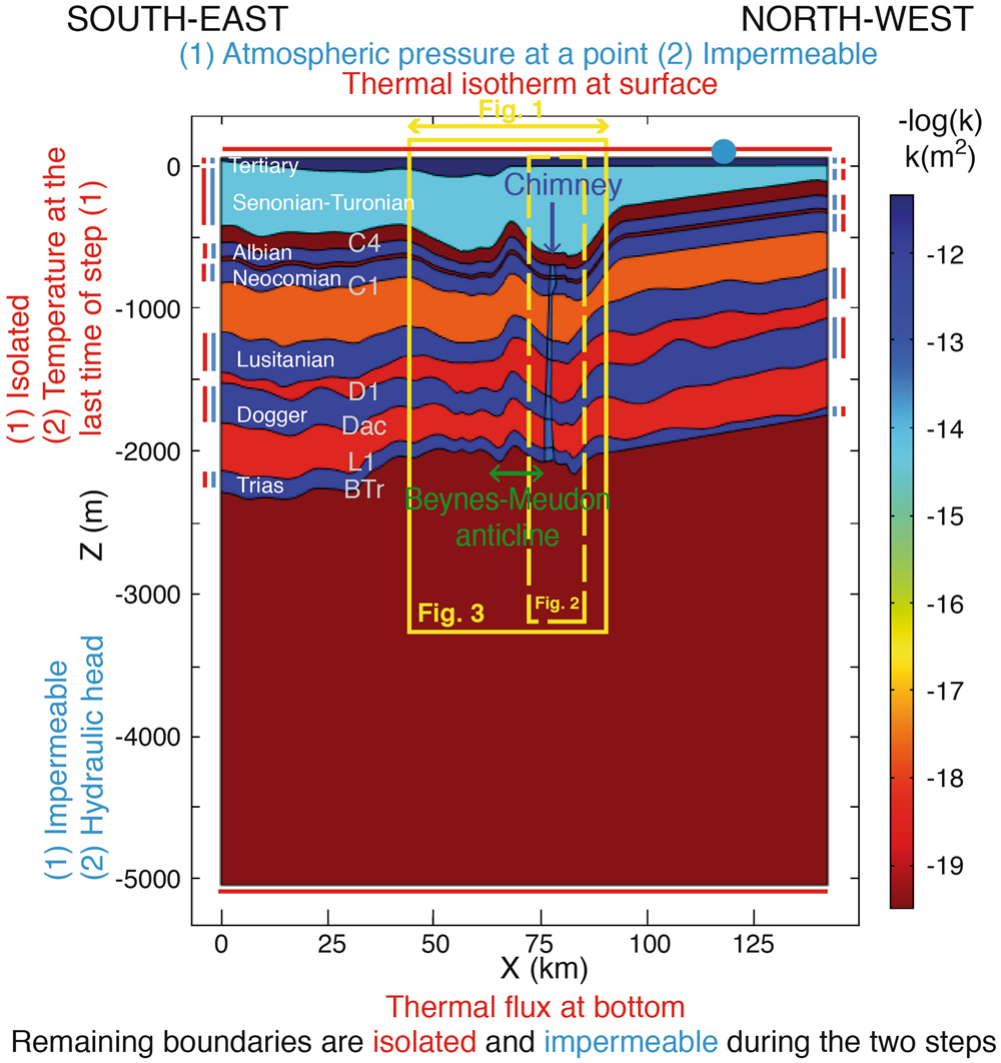
Geometry of cross-section, intrinsic permeability fields expressed in –Log(k) and hydrogeological and thermal conditions at boundaries during the stages of modelling. (1) first phase in steady-state regime termed “conductive”; (2) second phase in transient regime with mixed convection. The basal geothermal flux is 73 mW/m^2^ ^38^ and the surface temperature is 10.6 °C^23^. The yellow dashed and unbroken frames correspond to Figs 2 and 3, respectively.

The intrinsic permeabilities of aquifers interacting with the chimney were the subject of sensitivity studies. To simplify the sensitivity studies performed, these permeabilities were taken as identical. The value of 8.10^−13^ m^2^ retained for deep aquifers (Fig. 5 and Methods) may appear high.

However, the simulated hydrodynamic values can be discussed in the light of the data. The Darcy velocities obtained (Fig. 3) are close to those simulated by the hydrogeological models on the basin scale^31,32^. Although they are subject to discussion^31,32^, velocities around two orders of magnitude greater were also measured^33^ in comparison with these hydrogeological models. These velocities were measured in the producing environments of the Bathonian, which is the most sampled formation in the basin. Permeabilities there can be as high as 10^−11^ m^2^ ^29^.

The permeabilities of aquitards are consistent with the permeabilities used in Contoux *et al*.^25^, whose values are representative of a hydrogeological model on the basin scale.

The mixed-convective fluxes within the fractured zone depend on the combination of its permeabilities and thicknesses across aquifers and aquitards. As chimney widths are inherited from the seismic data, the method was to vary permeability. Best fitting of the model with the observed data was obtained by using continuous permeability between the aquifers and aquitards (Fig. 5, between kilometres 76 and 79). It is worth noting that the chimney to the north of Paris shows a disrupted area that is continuous between aquifers and aquitards (Fig. 2). A permeability of 5.10^−14^ m^2^ for this chimney was therefore finally adopted. This led to a local reduction of the aquifer permeabilities whereas these permeabilities are considered in a first approximation as homogeneous across the model and identical for the aquifers considered, which constitutes a simplified approach.

This permeability of the chimney is included in the values synthesised from the bibliography. Values range from 10^−14^ m^2^ to 10^−11^ m^2^ for numerical simulations of structures allowing passage of flows according to Cherubini^34^. However, they relate to faults, which are currently much more fully documented than chimneys.

Porosities^27–30^, specific storage coefficients^25^ and thermal conductivities^35,36^ for the formations are from the literature. The specific storage coefficient within the chimney is higher than in the surrounding environment, whereas its thermal conductivity is equal to the mean conductivity of the sedimentary formations.

Lastly, properties considered as homogeneous in the model are: the density *ρ* at 2,500 kg/m^3^, the heat capacity C_p_ at 1,230 J/kg/K, (these are means from Gaulier and Burrus)^37^, and the coefficients of longitudinal dispersivity *α*_1_ at 100 m and of transverse dispersivity *α*_t_ at 10 m.

### Stages of modelling and boundary conditions

The thermo-hydrogeological simulations (1 and 2) comprise two phases. Phase (1) – a steady-state regime without imposed flows and with lower intrinsic permeabilities than in the literature – was calculated first (Table 1) to obtain a conductive regime that initialises the transient regime. Phase (2) – a 5 Ma transient regime with a hydraulic head gradient imposed on the aquifers – was then calculated to attain a pseudo-equilibrium.

Where energy conservation is concerned, a surface temperature of 10.6 °C is imposed^23^ and a uniform thermal flux of 73 mW/m^2^ ^38^ is prescribed at the lower limit of the cross-section (Fig. 5).

Where groundwater flow is concerned, hydraulic heads are imposed for each aquifer. The oldest available measurements are used for the less disrupted hydrodynamic regime, i.e. without pumping. The hydraulic heads in the Albian are from the piezometric regime in 1930, which is referred to as “pre-pumping”^27^ (Table 1). The Neocomian is considered to have an equivalent hydrodynamic regime with an additional 20 m of head in relation to the Albian. For the Bathonian aquifer, the estimate is derived from the map from Rojas *et al*.^16^, which is not at equilibrium because of the pumping from the Albian^25^. For the Lusitanian, the hydraulic heads are intermediate between the values for the Bathonian and Neocomian. For the Triassic, the hydraulic head boundary conditions are estimated from the sparse measurements available^32^. Lastly, the hydraulic heads in the Chalk, from the piezometric regime described in the work of Raoult^27^, are not suited to the study. This study does not take into account the topography and surface hydrological network with the orientation of the cross-section constrained by flows in deeper aquifers. The hydraulic heads in the Tertiary, which are linked to the water levels in the river Seine, are not satisfactory for the same reasons (see Supplementary Information).

## Electronic supplementary material

Supplementary Information

## Data Availability

The data that support the findings of this study are available from the corresponding author upon duly motivated request.
