# Supplementary material for: Thermal and seismic hints for chimney type cross-stratal fluid flow in onshore basins
Source: Sci Rep. 2018 Oct 17;8:15330. doi: 10.1038/s41598-018-33581-x (PMC6192993; doi:10.1038/s41598-018-33581-x)
Supplement: Supplementary file 1 — Supplementary Information [file 41598_2018_33581_MOESM1_ESM.docx]

## Supplementary information

**Thermal and seismic hints for chimney type cross-stratal fluid flow in onshore basins**

Jacques Dentzer 1,2*, Dominique Bruel 3, Matthias Delescluse 1, Nicolas Chamot-Rooke 1, Laurent Beccaletto 4, Simon Lopez 4, Gabriel Courrioux 4, Sophie Violette 1,2

1 UMR 8538, Laboratoire de Géologie, Département de Géosciences, Ecole normale supérieure, PSL Research University / CNRS, 24 rue Lhomond, 75231 Paris Cedex 05, France, 2 UFR 918, UPMC-Sorbonne Universités, 4 place Jussieu, 75252 Paris Cedex 05, France, 3 Centre de Géosciences, Mines ParisTech, PSL Research University, 35 rue Saint Honoré, 77305 Fontainebleau, France, 4 Direction des Géoressources, BRGM, 3 avenue Claude Guillemin, BP 36009, 45060 Orléans Cedex 2, France. *e-mail: [jacques.dentzer@ens.fr](mailto:jacques.dentzer@ens.fr)

## Contents

Temperature and salinity data (incl. Fig. S1 and Fig. S2)

Seismic data (incl. Fig. S3, Fig. S4 and Fig. S5)

Choice of 2D cross-section alignment

GeoModeller import and simplifications

Modelling perspectives

Table S1


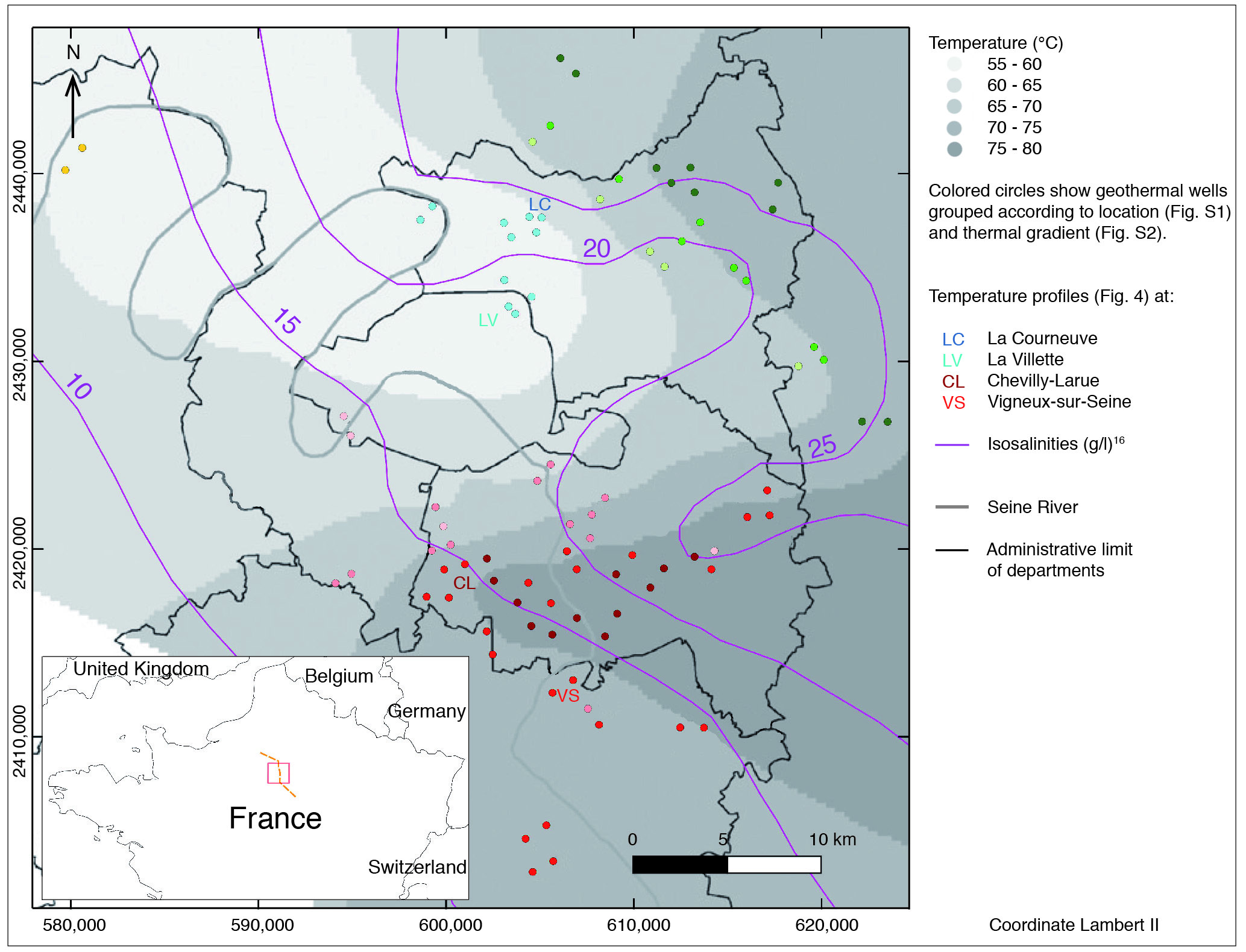
**Temperature and salinity data.**

**Figure S1.** Temperature and salinity in the Bathonian around Paris in the Anglo-Paris Basin. a, Overlaying of i) isotherms (modified from Lopez *et al*.^13^); ii) grouping of geothermal wells (Fig. S2); iii) temperature profiles illustrated in Figure 4; iv) isosalinities in the Bathonian (Dogger)^16^.


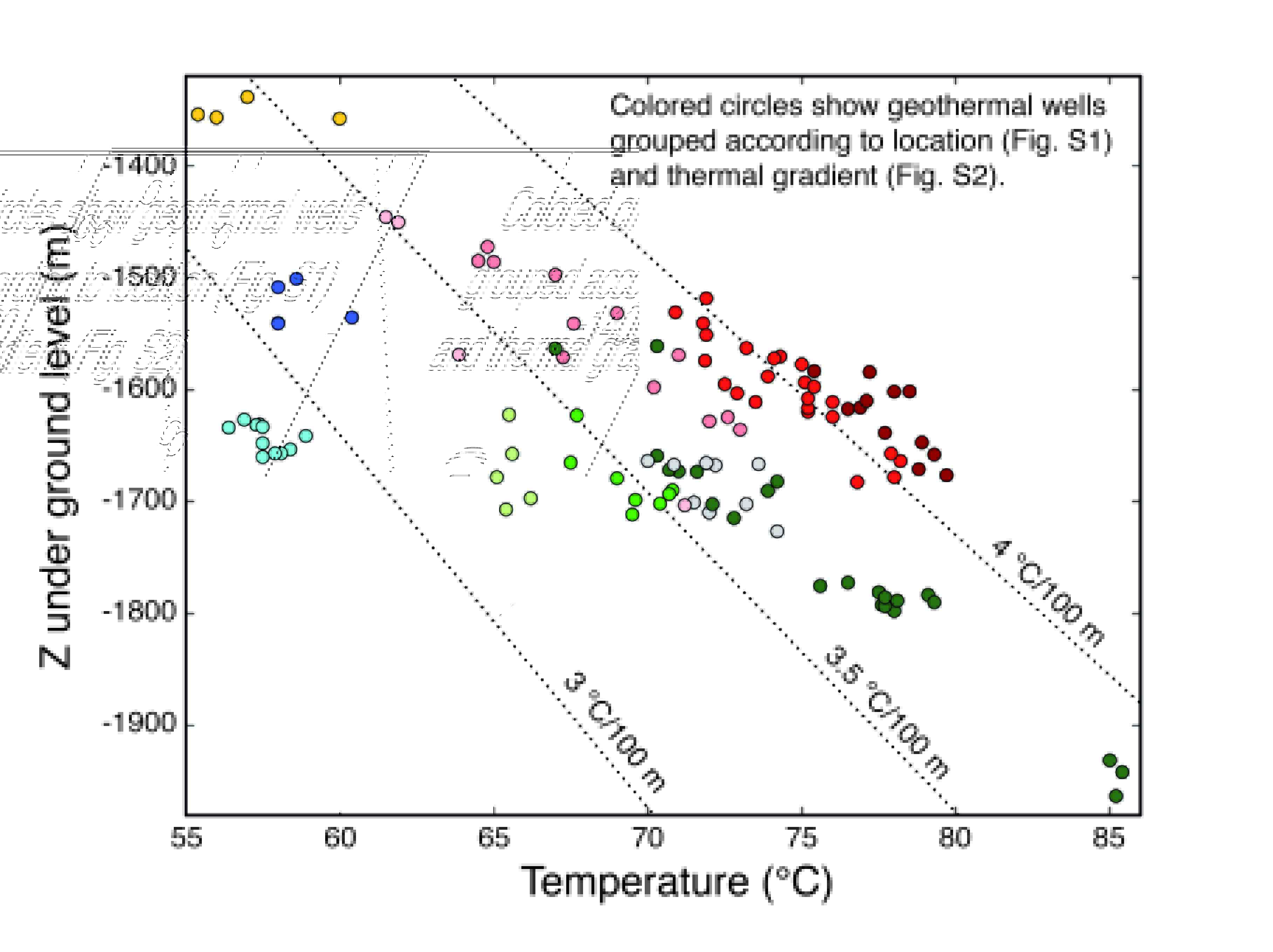
**Figure S2**. Temperature (°C) as a function of Z (m) for geothermal wells in the Bathonian in the Anglo-Paris Basin (Fig. S1) and alignments of temperature profiles with thermal gradients and 10.8°C at the surface.

**Seismic data.** Seven unpublished seismic lines, acquired between 1986 and 1991, were reprocessed (Fig. 1 and Fig. S3; 86PIF12, 86PIF10, 86PIF18, 85PA01, 87PIF01, 86PIF01, 86PIF11, 91PIF02). The total length of the seismic lines is 122.6 km.

**
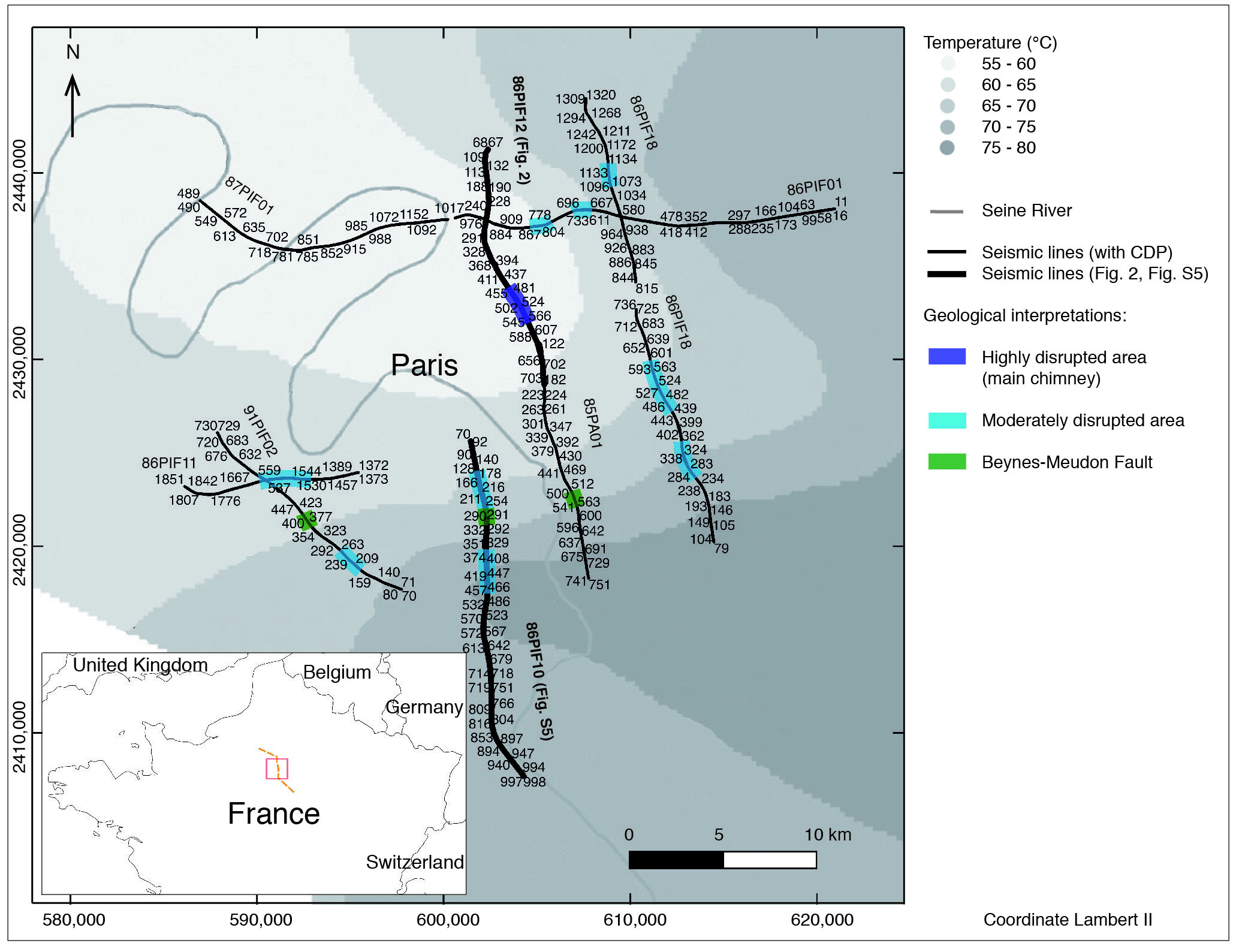
**

**Figure S3.** Seismic lines around Paris in the Anglo-Paris Basin (in black lines and thicker black lines where illustrated).

The geological model of the processing along line 86PIF12 is illustrated in Fig. S4. The near-surface was described by a detailed static model. It is based on a well-informed geological study. In addition, it is constrained by several boreholes with seismic velocities (Fig. S4). One of these wells is just above our main interpreted chimney (Fig. 2 and Fig. S4). Finally, we can see that despite many irregularities at the surface in other places, there are not always vertical noise trails/dimming zones in the reflection seismic data below.

The CDP fold of the data was investigated. It is fairly constant and, along all the profiles, there was no correlation between the slight CDP fold variations and the dim zones (for instance Fig. S4). Fold is constant (around 40) at the location of the main chimney (Fig. S4). Low-fold zones do not correlate with dimmer seismic amplitudes.


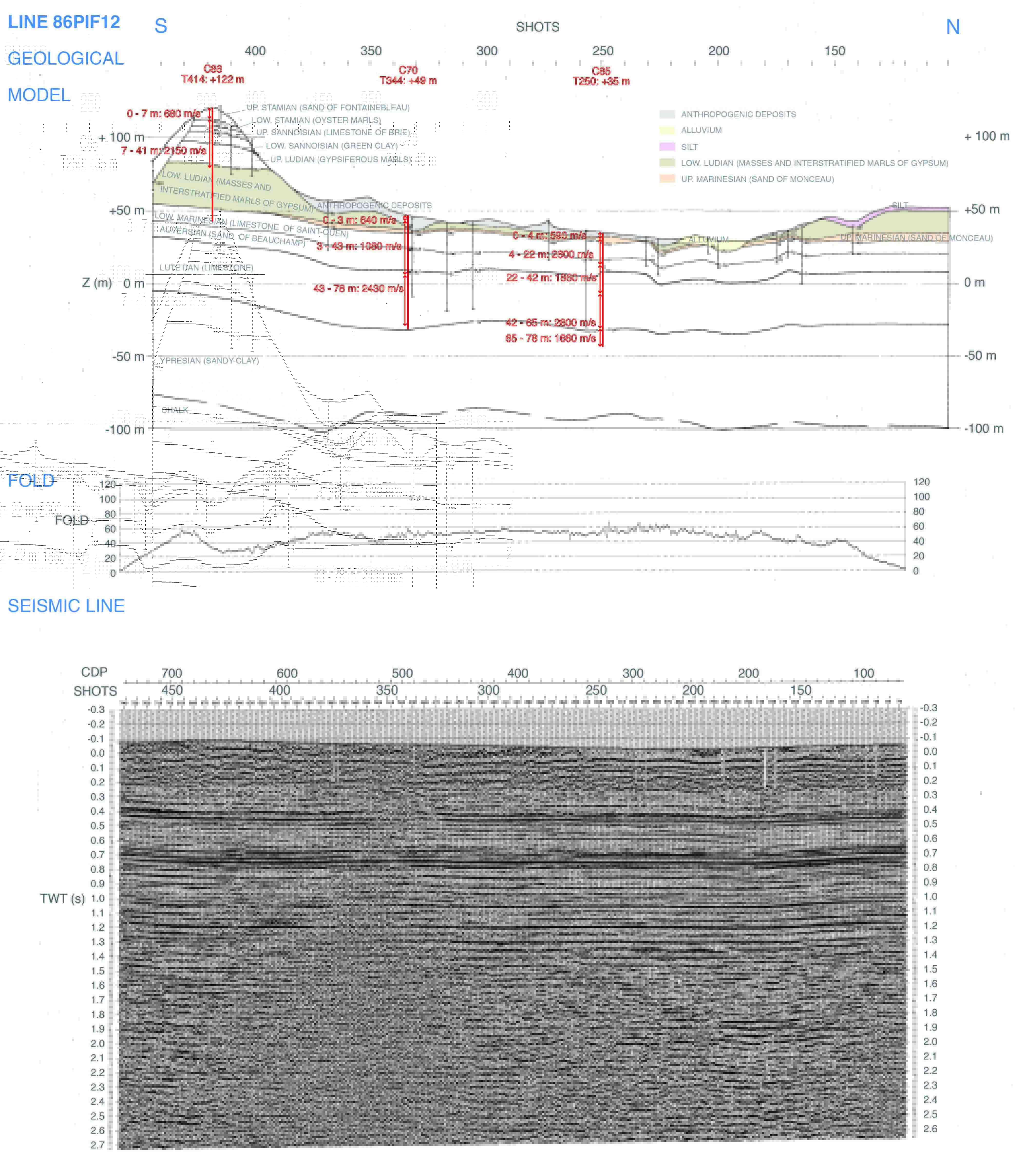


**Figure S4.** Geological model, fold and seismic line of 86PIF12.

The raw seismic data were reprocessed using a standard processing flow to produce final pre-stack migration data. The main profiles of interest are presented as a time section (Fig. 2 and Fig. S5). Their estimated vertical resolution is around 20 m.

Six seismic horizons have been interpreted and calibrated by using neighbouring geothermal and hydrocarbon wells: base of the Triassic formation (BTr), top of the Triassic (L1), top of the marls at Ostrea Acuminata marls (Dac), top of the calcareous Dogger (i.e. top of the Dalle Nacrée, D1), lower Berriasian/upper Berriasian limit (C1), and top of the Albian (C4). The terminology used throughout is based on Geofrance 3D^20^.


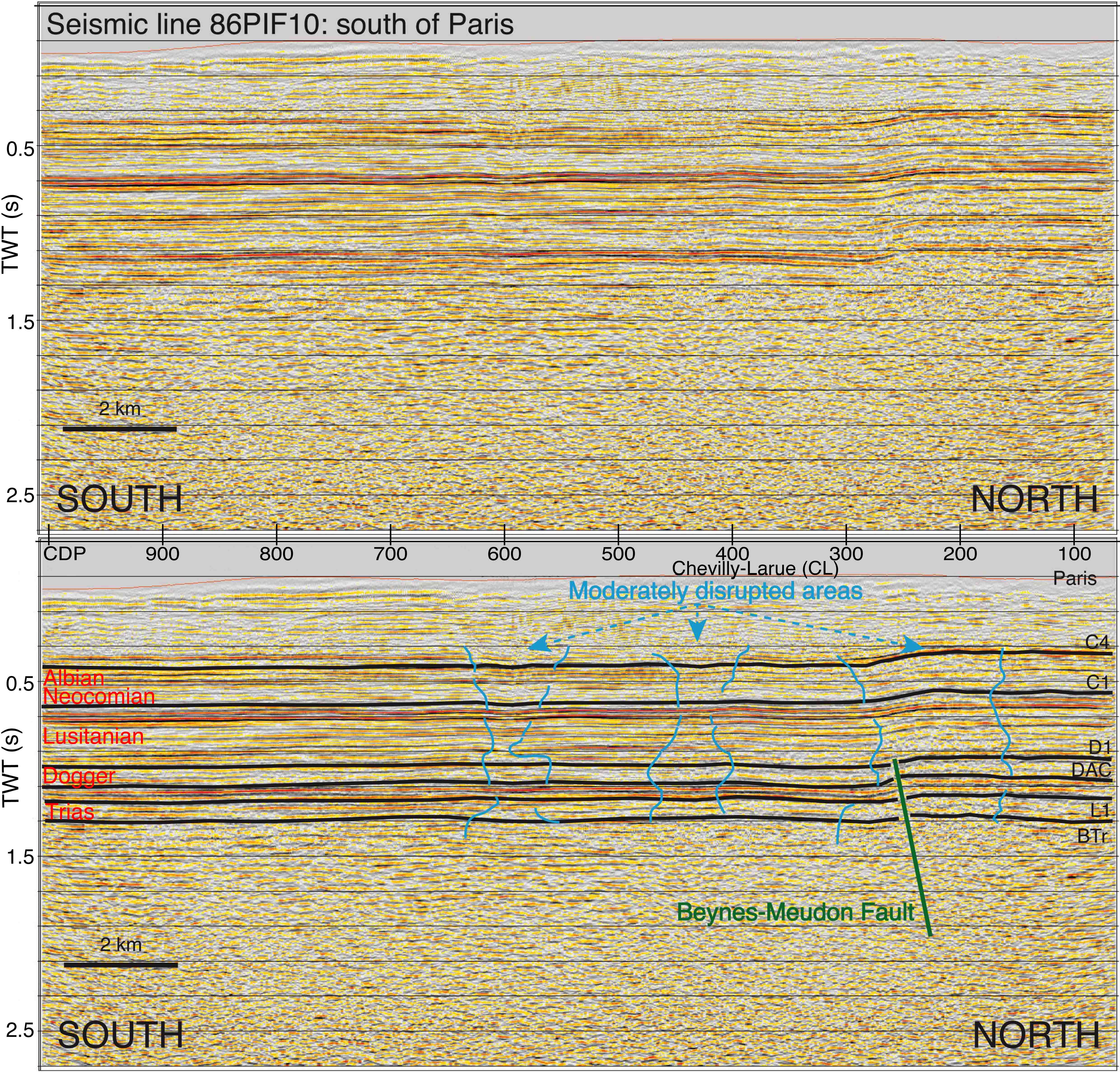


**Figure S5.** Interpreted north-south seismic line (86PIF10) to the south of Paris (Fig. 1 and S3). Locations of cities in black (Fig. 1 and 4). Observations of the Beynes-Meudon fault (in green) and areas with moderately disrupted seismic facies bounded by light blue lines. Main deep aquifers (in red). Geological interpreted horizons (in black): base of the Triassic (BTr); top of Triassic (L1); top of Ostrea Acuminata marls (Dac); top of calcareous Dogger (i.e. top of Dalle Nacrée, D1); lower Berriasian/upper Berriasian limit (C1); and top of Albian (C4).

**Choice of 2D cross-section alignment.** 2D simulations were chosen to describe the mixed convection in the formations and the chimney because of the computing time required. Figure 1 represents the location of the entire cross-section. This approach is analogous to that of other studies, for example Yang *et al*.^5^, Simms and Garven^4^, Magri *et al*.^6^.

The alignment of the 142 km cross-section (Fig. 1 and Fig. 5) is a compromise between different criteria: i) structural; ii) hydrogeological^25^ and iii) thermal. Priority is given to deep aquifers because of location of data on the Dogger. The alignment runs along the only line that allows observation to the north of Paris. The cross-section therefore passes through the area with the main disrupted seismic facies, at 79 km from the south-east boundary of the cross-section. It happens that the seismic line further to the east (e) is weak in terms of data acquisition at the same latitude. Interestingly, the geochemistry indicates a less saline area with an east-west orientation^16^ there.

**GeoModeller import and simplifications.** The GeoModeller® modelling tool^40,41^ was used to construct a 3D geological model of the centre of the Meso-Cenozoic basin from the Triassic to the Tertiary formations. The modelled horizons correspond to the stratigraphic markers of the Geofrance 3D work^20^ used by Jost^42^. However, the Triassic has been simplified to a single formation.

The 3D geological model is based on the following data:

1. slightly fewer than 500 wells, which are mainly from the Geofrance 3D work^20^ and geothermal wells database^29,43^;
2. the reinterpreted seismic lines in the centre of the Île-de-France region;
3. a numerical model of the terrain with lateral resolution of 1,000 m.

The geometry from GeoModeller is then simplified (Fig. 5), with the aquitards between aquifers being merged. In addition, the level of resolution of the resulting hydrogeological model is that of the geological formation (GeoFrance 3D, with the exception of the Triassic). It does not, therefore, take into account the set of aquifers making up the Tertiary formation nor the complex distributions of recharge and discharge areas on the surface. Moreover, this 2D study is concerned with deep aquifers, so the surface topography is assumed to be flat with a mean altitude of 60 m. For example, Fig. 5 shows a simplified and modified vertical cross section from this model.

**Modelling perspectives.** The aim was not to reproduce the temperatures exactly, all the more so since there are other phenomena present from the surface to the deeper horizons.

First, conductive phenomena can be considered. The non-integration of thermal phenomena at the air/ground interface could explain around 2°C ^44^ of discrepancy. Then, although first horizons are impacted by global warming, the Bathonian aquifer, which is our main target, is too deep to be affected currently by climate change. The oldest known air temperature in Paris, from the late 19th century, was therefore imposed. Other phenomena could, consequently, be palaeoclimatic ones^45,46^, for the deeper sedimentary horizons for example. Lastly, the presence of granites in the heat generating substratum^47^ could explain spatial heterogeneities in basal geothermal flux. However, these phenomena alone could not explain the geochemistry observed.

Second, mixed-convective phenomena can be considered for the thermal anomaly. The anomaly between the north and the south of Paris is recorded from the first horizons (Fig. 4). It is worth noting that this cannot be explained by spatial heterogeneities in basal geothermal flux. However, it underscores the advantage of a better description of the upper Cretaceous and Tertiary aquifers in the light of the topographical effects and links with the hydrographic network. For instance, there is the notable example of Boutonnière de Bray (north-west, off the main map in Fig. 1) and its potential for aquifer recharge^27^. In the north-east German basin, there is also the example of simulations by Cherubini^34^ that show cold anomalies due to downward recharge flows. To a depth of 1,800 m these anomalies are correlated to maximum topography. Rogiers *et al*.^48^ also describe a correlation between low thermal gradients and high topography in Belgium.

The 3D thermal functioning of the basin may include downward movement of fluid on the one hand, but also rising fluid on the other. Lower temperatures to the north of Paris beyond the first horizons (Fig. 4) could, in fact, be due to a local recharging area. A finer study of aquifers in the Tertiary, below the resolution of this model, would be required to confirm or deny this hypothesis. Then, saline fluid rising from the Triassic in the south^49^ could explain the warmer temperatures in comparison to the mean thermal trend. This argues for integration of processes in three dimensions. Moreover, a 3D model would better integrate heterogeneities such as chimneys in comparison with this first simple approach with a 2D model.

Integrating 3D thermo-haline mechanisms in chimneys is also a logical follow on to this work. This would allow verification of the viability of these mixed-convective flows i.e. convective cells. A limit on the validity of criteria for the appearance of convection in two dimensional models is that natural convection is a three dimensional process^4^. Then, the appearance of natural convection in a warmer more saline environment at depth could be limited by the competing co-existence of these two density related processes^5,32^. This limitation would reinforce the cold anomaly by a descending flow in a chimney more permeable than the one considered in this study. In addition, this would allow use of salinity as a tracer and additional means of constraining the model.

**Table S1.** Nomenclature.

| Symbol | Name | Units |
| --- | --- | --- |
| $X, Z$ | Spatial dimension | $m$ |
| $t$ | Time | $s$ |
| $\omega$ | Porosity | $\%$ |
| $\rho$ | Saturated matrix density | ${kg}/{m^{3}}$ |
| $\rho_{w}$ | Density of water | ${kg}/{m^{3}}$ |
| $g$ | Gravity | $m/{s^{2}}$ |
| $\theta$ | Temperature | $K$ |
| $C_{p}$ | Saturated matrix heat capacity | $J/{kg/K}$ |
| $C_{pw}$ | Heat capacity of water | $J/{kg/K}$ |
| $\lambda$ | Saturated matrix thermal conductivity | $W/{m/K}$ |
| $\boldsymbol{\lambda}_{\mathbf{disp}}$ | Macrodispersivity term | $W/{m/K}$ |
| $\boldsymbol{\lambda}_{\mathbf{eq}}$ | Equivalent thermal conductivity | $W/{m/K}$ |
| $\lambda_{w}$ | Thermal conductivity of water | $W/{m/K}$ |
| **φ** | Heat flux density | $W/{m^{2}}$ |
| $k$ | Intrinsic permeability | $m^{2}$ |
| μ | Dynamic viscosity | $Pa.s$ |
| $S_{s}$ | Specific storage coefficient | $m^{-1}$ |
| $p$ | Pressure | $Pa$ |
| $h$ | Hydraulic head | $m$ |
| $\mathbf{U}$ | Darcy velocity | $m/s$ |
| $\alpha_{l}$ | Longitudinal dispersivity | $m$ |
| $\alpha_{t}$ | Transverse dispersivity | $m$ |

**References (for Supplementary Information only)**

40. Lajaunie, C., Courrioux, G. & Manuel, L. Foliation fields and 3D cartography in geology: Principles of a method based on potential interpolation. *Math. Geol.* **29,** 571–584 (1997).

41. Calcagno, P., Chilès, J. P., Courrioux, G. & Guillen, A. Geological modelling from field data and geological knowledge: Part I. Modelling method coupling 3D potential-field interpolation and geological rules. *Phys. Earth Planet. Inter.* **171,** 147–157 (2008).

42. Jost, A. Caractérisation des forçages climatiques et géomorphologiques des cinq derniers millions d’années et modélisation de leurs conséquences sur un système aquifère complexe : le bassin de Paris. (Characterization of climatic and geomorphologic forcings of the last five million years and modelling of their effects on a complex aquifer system, the Paris basin.). (Université Pierre et Marie Curie - Paris VI, 2005).

43. Hamm, V. & Giuglaris, E. *Gestion du Dogger en Île-de-France*. 51 (BRGM, 2014).

44. Kitover, D. C., van Balen, R. T., Roche, D. M., Vandenberghe, J. & Renssen, H. New Estimates of Permafrost Evolution during the Last 21 k Years in Eurasia using Numerical Modelling. *Permafr. Periglac. Process.* **24,** 286–303 (2013).

45. Dentzer, J., Lopez, S., Violette, S. & Bruel, D. Quantification of the impact of paleoclimates on the deep heat flux of the Paris Basin. *Geothermics* **61,** 35–45 (2016).

46. Dentzer, J., Violette, S., Lopez, S. & Bruel, D. Thermal anomalies and paleoclimatic diffusive and advective phenomena: example of the Anglo-Paris Basin, northern France. *Hydrogeol. J.* **25,** 1951–1965 (2017).

47. Debeglia, N. *Réinterprétations géophysiques dans le centre du bassin de Paris pour l’évaluation du potentiel géothermique (COPGEN 2005)*. 59 (BRGM, 2005).

48. Rogiers, B., Huysmans, M., Vandenberghe, N. & Verkeyn, M. Demonstrating large-scale cooling in a Variscan terrane by coupled groundwater and heat flow modelling. *Geothermics* **51,** 71–90 (2014).

49. Dentzer, J. Environmental forcings and structural controls on the present thermal regime of the Paris Basin: issues for understanding the geothermal potential in Île-de-France. (Université Pierre et Marie Curie - Paris VI, 2016).
